# Understanding Public Willingness to Undergo Blood Testing for Alzheimer's Disease Diagnosis: A Cross‐Country Analysis

**DOI:** 10.1002/alz70858_103796

**Published:** 2025-12-24

**Authors:** Hannah Brown, Simone Gabriele, Georgia Giannakopoulou, Sandra Teoh, Joseph I Vindici, Nazira Jainis, Hasifullah Ibrahim, Amber King

**Affiliations:** ^1^ Ipsos, London, London, United Kingdom; ^2^ Ipsos, Kuala Lumpur, Kuala Lumpur, Malaysia; ^3^ Ipsos, New York, NY, USA; ^4^ Ipsos, Kuala Lumpur, Kuala Lumpur, United Kingdom

## Abstract

**Background:**

Blood‐based biomarker tests for Alzheimer's Disease (AD) are showing promise in clinical trials and are expected to improve the accuracy of diagnosis. By facilitating earlier detection, these tests could enable more individuals to qualify for recently approved disease‐modifying therapies (DMTs). This study assessed public willingness to undergo such blood tests.

**Method:**

An online survey was conducted in August 2024 with 804 adults aged 18 years or older from the US (*n* = 106), EU4+UK (France, Germany, Italy, Spain, UK; *n* = 517), Japan (*n* = 106), and China (*n* = 75). Respondents were screened to ensure some understanding of, but not currently diagnosed with, AD. Participants were presented with a hypothetical scenario in which they were experiencing symptoms of mild cognitive impairment (MCI). They rated their willingness to take a blood test to confirm whether these symptoms were due to AD on a 7‐point scale (1 = Not at all willing, 7 = Very willing). Ratings of 6 or 7 were considered “very willing” (Top 2 Box, T2B).

**Results:**

Overall, 53% of respondents indicated high willingness (T2B) to take the blood test. Willingness was highest in the US (66%) and lowest in Japan (27%) (*p* < 0.05).

Lower willingness reported in Japan may be influenced by a cultural tendency to avoid extreme responses in surveys.

Significant factors associated with higher willingness included better understanding of the disease, personal experience with AD, greater concern about developing AD, and closer proximity to support networks.

A significant association was also observed between willingness to undergo a blood test and subsequent willingness to take treatment if MCI were confirmed but symptoms were not yet impacting day‐to‐day life. Among the 427 individuals willing to take a test, 77% were very willing (T2B) to take treatment, compared to 39% among the 125 individuals not willing to take a test.

**Conclusions:**

In this study cohort, variations exist in willingness to undergo blood testing for AD confirmation among different subgroups. These findings suggest that personal experience and knowledge of AD play crucial roles in shaping attitudes toward diagnosis. Consequently, educational initiatives may need to be differentiated for certain subgroups.

